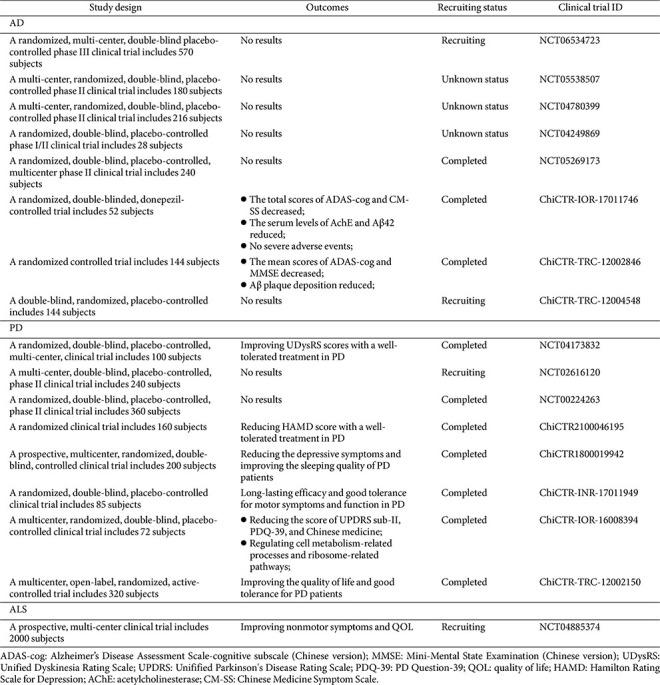# Chinese Herbal Medicine Targets Gut Microbiota to Combat Neurodegenerative Diseases: Potential Mechanisms and Clinical Implications

**DOI:** 10.4014/jmb.2510.10008

**Published:** 2026-02-25

**Authors:** Xi Yang, Yanling Qin, Wenxiu Mu, Wei Gao, Qi Chen, Jia Xia, Zhaozhao Huang

**Affiliations:** 1 Department of Neurology, Chengdu Pidu District Traditional Chinese Medicine Hospital, Chengdu, Sichuan 611731, P.R. China; 2 Department of Critical Care Medicine, Chengdu Pidu District Traditional Chinese Medicine Hospital, Chengdu, Sichuan 611731, P.R. China

**Keywords:** Neurodegenerative diseases, Chinese herbal medicine, Gut microbiota, Brain-gut-microbiota axis, Therapeutic mechanism

## Abstract

Due to the aging of the population, neurodegenerative diseases (NDs) have gradually become a major public health problem worldwide. Accumulating evidence has demonstrated that the gut microbiota and its metabolites were closely related to the occurrence and development of NDs. At present, Chinese herbal medicine (CHM) is known for its multi-dimensional, multi-target, and multi-pathway approach in the prevention and treatment of various diseases by regulating the gut microbiota, and different CHMs can regulate the diversity of gut microbiota and the abundance of probiotics. Modern studies have also revealed that CHM possessed therapeutic effects against NDs by targeting gut microbiota, regulating the secretion of neuroactive metabolites, reducing amyloid-beta deposition and oxidative stress, and improving the function of the blood-brain barrier. Therefore, the dynamic interaction among CHM, gut microbiota, and NDs has become a research hotspot. This review elaborates on the research progress related to CHM, gut microbiota, and NDs, aiming to provide a new perspective and theoretical basis for the prevention of NDs by CHM administration.

## Introduction

Neurodegenerative diseases (NDs), including Alzheimer’s disease (AD), Parkinson’s disease (PD), amyotrophic lateral sclerosis (ALS), Huntington’s disease (HD), and multiple sclerosis (MS), are chronic and progressive diseases of the nervous system. The presence of cognitive deficits and neuroinflammation is a key clinical feature of ND-related medical conditions [1, 2]. With aging populations worldwide, the prevalence of AD [3] and PD [4] is projected to be 13.8 million and 1.96 million by 2060 in the USA, imposing immense socioeconomic burdens. The World Health Organization estimates that NDs may become the second leading cause of death globally by 2040 [5], highlighting the urgency of exploring effective therapeutic strategies. In 2019, the direct healthcare expenditure for AD and related dementias (ADRD) reached $260.6 billion in 204 countries, and ADRD-related healthcare expenditure will reach $1.6 trillion by 2050 [6]. Current therapeutic strategies, primarily targeting protein misfolding (*e.g.*, amyloid-beta (Aβ) in AD) or neurotransmitter deficits (*e.g.*, dopamine in PD), offer symptomatic relief but fail to halt disease progression [7]. However, the pathogenesis and effective clinical treatment strategies for NDs remain elusive.

Accumulating evidence has demonstrated that the gut microbiota served as a pivotal modulator of central nervous system health, bridging intestinal ecology with neurodegenerative pathogenesis through the microbiota-gut-brain axis [8]. Previous studies have demonstrated that gut microbiota dysbiosis was detected in AD patients [9], PD patients [10], HD [11], ALS [12], and MS [13]. For example, increased relative abundances of *LachnospiraceaeUCG010*, *RuminococcaceaeUCG002*, *Clostridium sensustricto1*, *Eubacterium hallii* group, and *Bacillales* were associated with an elevated risk of PD, whereas higher *Bifidobacterium* abundance was inversely linked to PD susceptibility [14]. Zhao and colleagues [15] reported that increased *Bifidobacterium bifidum* levels were associated with a reduced risk of AD, but upregulated *Sutterellaceae* abundance correlated with an increased AD risk. Moreover, gut microbiota dysbiosis was correlated with cognitive impairment, motor complications, neuroinflammation, and major depressive disorder [16, 17]. ALS is characterized by reduced anaerobic bacteria (*e.g.*, *Eubacterium rectale*) and increased *Escherichia coli*, which may promote neuroinflammation [18]. Mechanistically, gut microbiota dysbiosis-driven metabolic perturbations (*e.g.*, short-chain fatty acid (SCFA) depletion, lipopolysaccharide (LPS) leakage, Trimethylamine-N-oxide (TMAO) and 5-hydroxytryptamine (5-HT) secretion) and immune activation underlie neurodegenerative cascades, including Aβ aggregation, tau phosphorylation, alpha-synuclein (α-SYN) misfolding, neuroinflammation [19]. Meanwhile, modulation of gut microbiota by exercise, medicinal herbs, and dietary intervention can alleviate the progression of NDs [20, 21]. Fecal microbiota transplantation exhibited beneficial effects on neurological symptoms in NDs [22], which was safe and tolerable in the treatment of patients with MS [23] and ALS [24]. Zheng *et al*. [25] reported that probiotic *Clostridium butyricum* supplementation improved obesity-associated cognitive impairments and neurodegeneration by preventing gut microbiota dysbiosis, inflammation, and intestinal barrier impairment. Of note, gut microbiota composition and its metabolites can serve as a potential predictive biomarker for patients with ND [26, 27]. Collectively, targeting gut microbiota may be an effective approach for the prevention and treatment of ND.

Chinese herbal medicine (CHM), with its millennia of empirical clinical experience, possesses multi-faceted pharmacological activities with low toxicity, including anti-inflammatory, antioxidant, antitumor, and immunomodulatory properties [28]. Pharmacological studies have confirmed that CHM exhibited remarkable efficacy and favorable safety profiles in the prevention and treatment of NDs [29, 30]. Randomized controlled trials reported that adjunctive CHM therapy improves cognitive scores and motor function in ND patients, correlating with restored microbial diversity and reduced pro-inflammatory cytokines (*e.g.*, IL-6, TNF-α) [31, 32]. Functional, CHM possessed a beneficial effect against neurodegeneration by regulating the gut microbiota and its metabolites. For example, Gegen Qinlian formula administration reduced cognitive dysfunction and neuroinflammation of AD rats by remodeling gut microbiota homeostasis, as evidenced by enhancing the *Firmicutes*/*Bacteroidetes* ratio and SCFAs-producing taxa (*e.g.*, *Ruminococcaceae* and *Oscillospira*) [33]. Ginsenoside CK treatment suppressed neuroinflammation, oxidative stress, and the apoptosis of dopaminergic neurons in PD model mice by increasing the abundance of probiotics (*Bacteroides*) and decreasing the number of pathogenic bacteria (*Actinomycetes*) [34]. Oxymatrine treatment diminished inflammation and blood-brain barrier disruption by correcting gut microbiota dysbiosis and downregulating the levels of isobutyric acid and isovaleric acid in MS model mice [35]. Moreover, the gut microbiota contributed to enhancing the bioavailability and therapeutic efficacy of CHM in NDs by modulating its intestinal biotransformation and absorption. For instance, berberine administration ameliorated PD through enhancing brain dopa/dopamine levels mediated by the gut bacteria (*e.g.*, *Enterococcus faecalis* or *Enterococcus faecium*) [36]. Oral administration of piperine improved the move disorders of PD model mice by suppressing gut bacterial tyrosine decarboxylation [37]. Taken together, the therapeutic effect of CHM on various NDs by regulating the structure and metabolism of gut microbiota and maintaining intestinal homeostasis has gradually become a hot topic.

Herein, this review critically examines the involvement of gut microbiota in the pathogenesis and progression of NDs. In addition, this review synthesizes evidence supporting the neuroprotective potential of CHM on NDs by targeting the gut microbiota. Furthermore, the current clinical evidence base regarding the therapeutic efficacy and safety of CHM interventions for NDs is appraised, alongside a discussion of prevailing methodological challenges and promising avenues for future research.

## Methods

A comprehensive literature search was conducted in English databases (Web of Science, PubMed, ScienceDirect, Wiley, and Springer Nature). All published data till the year 2025 have been taken into consideration. The following search keywords were used in the search of materials for this study: ‘Chinese herbal medicine’, ‘Traditional Chinese medicine’, ‘natural product’, ‘active ingredients’, ‘polyphenols’, ‘flavonoids’, ‘alkaloids’, ‘terpenes’, ‘gut microbiota’, ‘biological activity’, ‘neurodegenerative diseases’, ‘Alzheimer’s disease’, ‘Parkinson’s disease’, ‘amyotrophic lateral sclerosis’, ‘Huntington’s disease’, ‘Multiple sclerosis’, and ‘polysaccharide’. Chinese databases were not searched. The studies providing the details of microbiome alterations in ND and of the CHM regulating microbiota were included. Literature containing incomplete references and issue number were excluded.

## Overview of Gut Microbiota

The gut microbiota, a complex ecosystem of commensal microorganisms residing in the human gastrointestinal tract, is mainly composed of microorganisms such as bacteria, viruses, archaea, and fungi [38]. At the phylum level, the gut microbiota is predominantly constituted of *Bacteroidetes*, *Firmicutes*, and *Proteobacteria*, while key genera include *Bacteroides*, *Prevotella*, *Faecalibacterium*, *Roseburia*, *Lachnospira*, and *Sutterella* [39].

Through millennia of co-evolution, the human host offers nutrients and a stable niche for the gut microbiota, while the gut microbiota reciprocally regulates host physiological functions. Beyond facilitating energy harvest and nutrient absorption, the gut microbiota critically modulates immune system maturation through antimicrobial peptide secretion (*e.g.*, defensins), pathogen displacement (*e.g.*, colonization resistance), and tight junction reinforcement (*e.g.*, mucin glycosylation). However, this delicate ecosystem is highly susceptible to dysregulation by exogenous factors, including dietary patterns, environmental toxins, and antibiotic overuse. Perturbations such as chronic inflammation, enteric infections, or xenobiotic exposure can cause dramatic intestinal dysbiosis, characterized by variations in the composition of gut microbiota and their corresponding metabolites, thereby triggering the occurrence and progression of diseases. Previous studies have found that gut microbiota dysbiosis contributed to triggering intestinal permeability and systemic inflammation [40, 41]. Moreover, gut microbiota served as a valuable diagnostic biomarker and therapeutic target across multiple pathologies [42, 43]. *Megamonas*, *Blautia*, *Pseudomonas*, *Stenotrophomonas*, and *Veillonella* were reported as potential novel biomarkers for older AD patients with cognitive impairment [44]. Notably, gut microbiota dysbiosis has been strongly associated with neurodegenerative disorders via the gut-brain axis, with altered microbial metabolites (*e.g.*, SCFAs and TMAO) influencing neuroinflammation and amyloidogenesis. For instance, administration of SCFAs ameliorated AD by reducing the Aβ burden and improving the blood-cerebrospinal fluid barrier [45]. A recent study showed that oral administration of *Akkermansia muciniphila* alleviated PD by inhibiting neuroinflammation and the reduction of dopaminergic neurons [46]. Consequently, maintaining microbial homeostasis, such as probiotics, fecal microbiota transplantation, personalized dietary interventions, and targeted drug therapies, is gaining traction as novel therapeutic strategies for various diseases.

## The Relationship between Gut Microbiota and Neurodegenerative Diseases

Accumulating evidence has indicated that the gut microbiota was a critical modulator in the pathogenesis of NDs [47]. Firstly, gut microbiota dysbiosis contributed to the occurrence and progression of NDs. In AD, fecal metagenomics revealed that both the total abundance and diversity of the gut microbiota were reduced in AD patients compared with healthy controls [48]. Zhuang *et al*. [9] reported that depletion of *Bacteroides*, *Actinobacteria*, and *Ruminococcus* was correlated with cognitive decline and microglial hyperactivation. Chen *et al*. [17] confirmed that gut microbiota dysbiosis was associated with cognitive impairment and microglial activation in AD patients. Liu *et al*. [49] reported that the clinical severity scores of AD patients were related to the abundance of altered microbiomes, especially the enrichment of the abundance of the family *Enterobacteriaceae*. Another study showed that *Eubacterium fissicatena* was associated with a reduced risk of AD development, whereas *Collinsella* and *Veillonella* were linked to increased disease susceptibility [50]. In PD, a meta-analysis showed that *Bifidobacteriaceae*, *Ruminococcaceae*, *Rikenellaceae*, *Lactobacillaceae*, *Verrucomicrobiaceae* and *Christensenellaceae* were found to have increased ratios according to the pooled ratios, while *Prevotellaceae*, *Lachnospiraceae*, *Erysipelotrichaceae* and *Faecalibacterium* were decreased in PD cases [51]. Takahashi *et al*. [16] showed that the abundance of *Lachnospiraceae blautia* and *Lactobacillaceae bactobacillus* was decreased and increased in PD patients, which were independent risk factors for motor complications. In patients with ALS, the abundance of *Escherichia coli* was significantly higher than that in the control group, while the abundance of *Anaerobic bacteria*, *Eubacterium rectale*, and *Megamonas* were significantly decreased [52]. HD patients present increased α/β-diversity and taxon-specific shifts compared to those in healthy controls [11]. Similarly, other studies have reported that the imbalance in gut microbiota composition has an impact on the symptoms and evolution of MS [53] and spinocerebellar ataxia [54]. Zeng *et al*. [55] reported the presence of gut microbiota dysbiosis and lack of SCFAs in Chinese MS, which was related to the immune function of patients. Zancan *et al*. [56] showed that decreased abundance of *Akkermansia muciniphila* was associated with an enhanced risk of MS, while increased *Ruminococcus torques* abundance emerged as a risk factor for MS. Moreover, dysbiosis of the gut microbiota not only served as diagnostic biomarkers but also predicted ND progression. Herein, the alterations in the composition of the gut microbiota and their roles in NDs are summarized in Table 1.

**Fig. 1:**
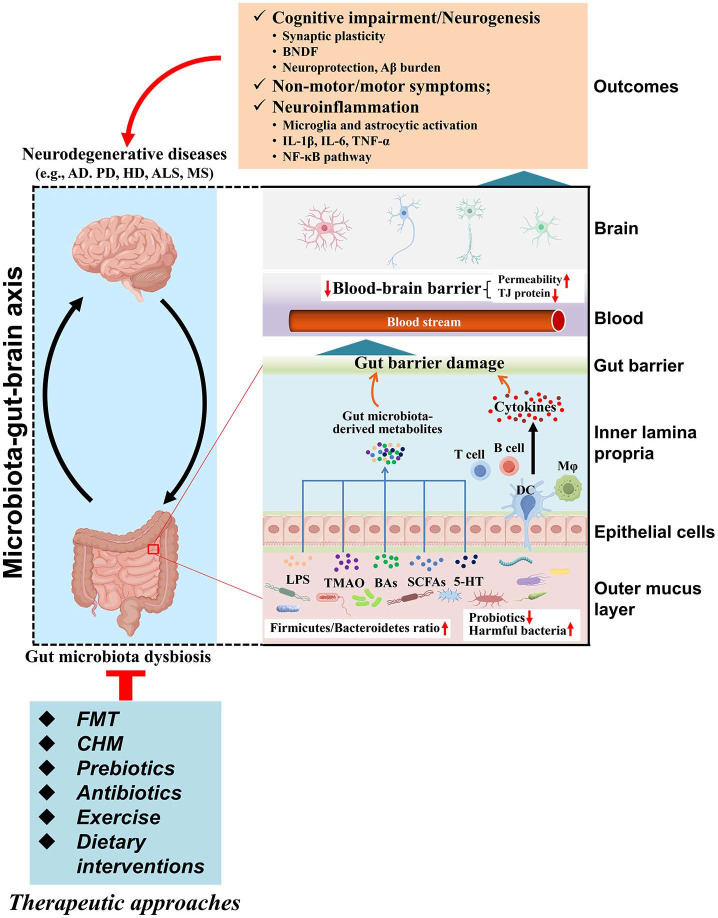
The functional role of the microbiota-gut-brain axis in neurodegenerative diseases. The gut microbiota influences the progression of ND through small molecule metabolites and breakdown products. In ND patients, dysbiosis of the gut microbiota alters metabolite and breakdown product levels, which can modulate neuroinflammation, affect BBB permeability, regulate ND pathology in the brain, and impact cognitive function. Treatments aimed at improving gut microbiota include FMT, CHM prebiotics, antibiotics, exercise, and dietary therapy. These therapies alleviate neuroinflammation and ND pathology through various mechanisms, improving brain Aβ deposition, BNDF, Tau phosphorylation levels, and BBB. ND: neurodegenerative disease; AD: Alzheimer's disease; PD: Parkinson's disease; HD: Huntington’s disease; ALS: amyotrophic lateral sclerosis; MS: multiple sclerosis; BNDF: brain-derived neurotrophic factor; IL: interleukin; TNF: tumor necrosis factor; LPS: lipopolysaccharide; TMAO: Trimethylamine-N-oxide; BA: bile acid; SCFA: short-chain fatty acid; 5-HT: 5- hydroxytryptamine; FMT: fecal microbiota transplantation; CHM: Chinese herbal medicine.

Functionally, gut microbiota dysbiosis was associated with the occurrence and progression of NDs through the gut-brain axis (Fig. 1). Meta-analysis of shotgun metagenomes delineates PD-associated microbial pathways potentially contributing to gut health deterioration and favoring the translocation of pathogenic molecules along the gut-brain axis [57]. Numerous studies have demonstrated that gut microbiota-derived metabolites (*e.g.*, SCFAs, bile acids (BAs), LPS, and TMAO) driven Aβ plaque load, neuroinflammation, neurodegeneration, microglia and astrocytic activation, as well as blood-brain barrier disruption [58-61]. Xie *et al*. [45] reported that supplementation with SCFAs improved the blood-cerebrospinal fluid barrier and reduced the Aβ burden in AD model mice. Quan *et al*. [62] showed that TMAO promoted PD progression by enhancing neuroinflammation and motor dysfunction, similar to the results of Qiao *et al*. [63]. *Bacteroides ovatus*-derived metabolite lysophosphatidylcholine reduced Aβ load and improved cognitive impairment in AD model mice by inhibiting ferroptosis [64]. Meanwhile, gut microbiota dysbiosis resulted in the impairment of the intestinal mucosal barrier, which accelerated neuroinflammation [65]. Other studies have found that gut microbiota-derived metabolites may serve as a pivotal factor in the pathogenesis of NDs [66, 67]. Additionally, the inflammatory responses of certain microbiota may also be one of the mechanisms influencing the pathogenesis of NDs. For example, oral pathogens such as *Veillonella parvula* and *Streptococcus mutans* exacerbated neurodegeneration and motor dysfunction by activating Th1-mediated neuroinflammation in PD models [68]. Another study has shown that Gram-positive segmented filamentous bacteria can promote gut microbiota dysbiosis, immune cell migration, intestinal inflammation, and α-SYN aggregation as well as motor deficits in AD model mice [69].

Importantly, therapeutic targeting of the gut microbiota holds promise for alleviating the progression of NDs. For example, preclinical studies have demonstrated that fecal microbiota transplantation can correct gut microbiota dysbiosis in PD model mice and inhibit gut and brain inflammation mediated by the LPS-TLR4 signaling pathway [70]. Gubert *et al*. [71] showed that fecal microbiota transplantation alleviated HD by ameliorating cognitive deficits and improving gut dysbiosis. Another study showed that supplementation with *Lactobacillus plantarum* DP189 can alleviate the progression of PD by reducing oxidative stress, inflammatory responses, and α-SYN deposition [72]. A randomized controlled clinical trial showed that transplanting the gut microbiota of healthy individuals into ALS patients can relieve the symptoms of ALS patients by increasing the number of T-regulatory lymphocytes [73]. Moreover, a systematic review and meta-analysis revealed that improving gut microbiota dysbiosis by probiotic supplementation, fecal microbiome transplant, antibiotics, dietary interventions, CHM treatment, and exercise can also prevent and manage NDs [74].

## Mechanism of Chinese Herbal Medicine in Preventing and Treating Neurodegenerative Diseases by Regulating Gut Microbiota

The global burden of NDs has escalated in parallel with aging populations, yet no disease-modifying therapies exist to halt or reverse their progression. This unmet medical need imposes substantial socioeconomic burdens on patients, families, and healthcare systems. Emerging evidence has confirmed that metabolic disorders and inflammatory responses caused by gut microbiota dysbiosis can be transmitted to the brain through the microbiota-gut-brain axis, potentially contributing to the pathogenesis of NDs [75]. Modern pharmacological studies have demonstrated that CHM (*e.g.*, CHM components, extracts, and formulas) exhibited therapeutic effects against NDs by regulating the structure and metabolism of gut microbiota and modulating body immunity [76, 77].

### Regulation of Gut Microbiota Composition

Emerging evidence has demonstrated that some CHM treatments exerted prebiotic-like effects analogous to prebiotics by enriching symbiotic beneficial bacteria (*e.g.*, *Bacteroides*, *Bifidobacterium*, and *Lactobacillus*), thereby restoring and maintaining physiological homeostasis [78]. Mechanistically, CHM treatment alleviated the progression of NDs by correcting the composition of the gut microbiota. For example, administration of Huanglian Jiedu decoction reduced cognitive impairment, neuroinflammation, and lipid metabolism disorder in APP/PS1 mice by increasing the abundances of *Prevotellaceae*, *Lactobacillaceae*, *Peptococcaceae*, *Alcaligenaceae*, and *Helicobacteraceae* and reducing the abundances of *Bacteroidales_S24-7*_group, *Lachnospiraceae*, and *Porphyromonadaceae* [79]. Ping-wei-san treatment enhanced the abundances of *Firmicutes* and *Verrucomicrobiota* and reduced the abundances of *Bacteroidota*, *Proteobacteria*, *Campilobacterota*, and *Patescibacteria* in PD model mice [80]. Heshouwu intervention increased the diversity and abundance of the gut microbiota in MS model mice [81]. Moreover, CHM extracts possessed anti-NDs by altering gut microbiota composition. For instance, *Poria cocos* extract treatments ameliorated the cognitive impairment of AD by improving gut dysbiosis, as evidenced by downregulated the abundances of *Bacteroidaceae*, *Lachnospiraceae*, *Ruminococcaceae*, *Rikenellaceae*, *Enterobacteriaceae*, and *Deferribacteraceae* and upregulated the abundances of *Muribaculaceae* and *Lactobacillaceae* [82]. Lu *et al*. [83] showed that aqueous ethanol extract of *Acanthopanax senticosus* improved the movement disorder of PD model mice by increasing *Firmicutes* and reducing *Actinobacteria* at the phylum level. Furthermore, single CHM components can attenuate the symptoms of NDs by improving gut microbiota dysbiosis. Icariin, an active ingredient extracted from *Epimedium* species, was shown to enhance the abundance of *Akkermansia* and reduce the abundance of *Alistipe* in APP/PS1 mice [84]. Gan *et al*. [85] reported that *Gastrodia elata* polysaccharide improved the motor dysfunction and reduced neuroinflammation of PD mice by elevating the levels of *Bacteroidetes* and reducing the levels of *Firmicutes* and *Verrucomicrobia*. Herein, the functional role of CHM (formulas, extracts, and compounds) in NDs by regulating gut microbiota is summarized in Table 2.

### Regulation of Gut Microbiota Metabolites

Numerous studies have demonstrated that CHM possessed a therapeutic effect against ND through the regulation of gut microbiota-derived metabolites [85, 86]. SCFAs, key metabolic byproducts of gut microbial fermentation, exert pivotal effects on inflammation modulation and intestinal mucosal homeostasis [87]. Previous studies have confirmed that dysregulated SCFA profiles (including acetic acid, propionic acid, and butyric acid) were detected in NDs, which was correlated with the altered gut microbiota composition [88, 89]. Preclinical studies have demonstrated that CHM treatment contributed to improving cognition impairment, reducing neuroinflammation, and enhancing blood-brain barrier integrity in ND model mice by increasing the contents of SCFAs [35, 90, 91]. Fu *et al*. [92] showed that administration of *Dendrobium officinale* polysaccharide reduced Aβ plaque deposition and restored intestinal barrier integrity in AD model mice by increasing SCFA content. Of note, SCFAs were reported to be an effective marker of gut dysbiosis in patients with AD [93]. Moreover, the protective effect of CHM on ND by reducing the levels of neurotoxic metabolites (including TMAO and LPS) derived from gut microbiota dysbiosis [94, 95]. Currently, dysregulation of TMAO levels linked to blood-brain barrier disruption, Aβ aggregation, α-SYN aggregation, and neuroinflammation [96] as well as LPS contributed to triggering microglia activation and neuroinflammation [97]. Other studies have demonstrated that CHM administration improved depressive behaviors in PD model mice by modulation of gut microbial tryptophan metabolism, as evidenced by upregulating the expression of 5-HT and brain-derived neurotrophic factor [98]. Furthermore, targeting of gut microbiota-derived metabolites BA by CHM has emerged as an effective therapeutic strategy for NDs. For instance, dioscin, a natural steroidal saponin, prevented neuroinflammation and oxidative stress in PD model mice by restoring gut dysbiosis and increasing BA levels [99]. *Poria cocos* extract treatments ameliorated pathological damage and reduced neuroinflammation and Aβ deposition in AD mice by reversing the metabolite dysfunction of BAs [82]. The above studies indicated that CHM exhibited a protective effect against NDs by modulation of gut microbiota and its metabolites, whereas the mechanistic underpinnings of CHM's regulatory effects on microbiota-metabolite crosstalk remain insufficiently explored, with limited experimental validation of causal relationships between specific microbial taxa, metabolic pathways, and neuroprotective outcomes. Herein, Table 2 summarizes the detailed information of CHM combated ND by regulating gut microbiota-derived metabolites.

### Regulation of the intestinal barrier function

Intestinal barrier dysfunction was associated with the pathogenesis of NDs. Previous studies have demonstrated that metabolic dysregulation compromises intestinal epithelial barrier integrity and permeability by impairing the expression and function of tight junction proteins (*e.g.*, ZO-1, occludin) and adherens junction proteins (*e.g.*, E-cadherin) [100]. Pathological hallmarks such as aberrant epithelial cell differentiation and downregulation of junctional proteins further exacerbate barrier defects, creating a vicious cycle that amplifies metabolic disturbances and ND progression [101]. CHM counteracts these effects by enriching beneficial gut microbiota (*e.g.*, *Bifidobacterium*, *Lactobacillus*) [102, 103], which in turn enhance intestinal barrier integrity via upregulation of mucin-2 secretion and junctional protein expression. This microbial remodeling reduces the translocation of pathobionts (*e.g.*, *Proteobacteria*) and neurotoxic metabolites (*e.g.*, LPS) across the gut-vascular barrier, thereby attenuating neuroinflammation and neuronal damage [104]. Chen *et al*. [105] showed that heteropolysaccharide from *Ganoderma lucidum* reduced the abundance of *Oscillibacter*, which was positively correlated with intestinal permeability. Other studies have found that administration of some CHM components (*e.g.*, DA-9601 [106] and icariin [84]) reduced intestinal epithelium injury and enhanced intestinal integrity by upregulating the abundance of *Akkermansia muciniphila*. Moreover, the active anti-ND compounds in CHM, such as berberine [103], sinomenine [107], *Polygonatum sibiricum* polysaccharides [108], *Gastrodia elata* polysaccharides [85], and *Dendrobium officinale* polysaccharides [92], repaired the intestinal barrier dysfunction in NDs by increasing the expression of the intestinal tight junction proteins. Similarly, the protective effect of CHM formulas on intestinal barrier dysfunction was also confirmed, including KaiXinSan-JiaWei [86] and Huanshaodan [109]. See Table 2 for details.

## Clinical Trials of Chinese Herbal Medicine for Neurodegenerative Disease Management

A large number of preclinical studies have proven the neuroprotective effects of CHM on ND by regulating gut microbiota. Currently, several randomized controlled trials have been performed to analyze the effectiveness/safety of CHM on NDs. For example, a randomized, double-blind, placebo-controlled trial demonstrated that administration of Tianqi Pingchan granule significantly reduced Unified Dyskinesia Rating Scale (UDRS) scores and the incidence of adverse events (4%) in PD patients compared with the placebo group, with the treatment exhibiting good tolerability [110]. Similarly, AD patient treatment with CHM obtained satisfactory clinical outcomes, such as Di-Tan decoction [111], Yishen Huazhuo decoction [112], Yokukansan [113], Huannao Yicong formula [114], and Kami-guibi-tang [115]. Li *et al*. [116] also reported that Bu-Shen-Jian-Pi formula was shown to reduce the rate of progression of SLC and side effects. Gu *et al*. [117] showed that treatment with Pingchan granule not only reduced UDRS scores in PD patients but also improved depressive symptoms. Meanwhile, Chinese medicine Bushen capsule exhibited long-term therapeutic effects against AD [118]. A 36-week multicenter, randomized, double-blind, placebo-controlled, parallel-group, phase 3 clinical trial showed that GV-971 (marine-derived oligosaccharide) treatment was safe and well-tolerated in AD patients, with significant improvements in cognitive function and sustained therapeutic effects [119]. Moreover, the combination of CHM with Western medicine (*e.g.*, donepezil or pramipexole) possessed synergistic benefits, including improved clinical outcomes [120], reduced hospitalization costs and adverse events [121, 122]. Another study reported that Bushen Yinao pill combined with conventional therapy enhanced cognitive function, reduced neuroinflammation, and improved gut microbiota dysbiosis of PD patients compared with the conventional therapy group [123]. These clinical trials suggested that CHM treatment can improve the quality of life and enhance non-motor function and cognitive capability of patients with NDs with a low incidence of adverse effects. Herein, ongoing national clinical trials evaluating CHM safety and efficacy in ND are summarized in Table 3 and supplementary Table 1.

## Conclusion and Perspectives

Given the continuous increase in the global prevalence of NDs and the limitations of existing therapeutic approaches, exploring new therapeutic targets is imperative. With the deepening of modern research, the bidirectional communication of the microbiota-gut-brain axis has been gradually revealed, and the gut microbiota plays an important role in the etiology and pathogenesis of NDs. Numerous studies have demonstrated that CHM treatment can be successfully used to treat NDs by targeting the gut microbiota, which is attributed to its pleiotropic effects, systems-level actions, multi-mechanistic characteristics. In addition, dietary strategies based on improving the gut microbiota also exert protective effects in ND models, providing solid experimental evidence for the basic theory of CHM tonic.

However, the relevant mechanisms of CHM targets “gut-brain axis” to combat NDs have not been fully elucidated, and many issues still need to be deeply explored: (1) Whether the gut microbiota can be used as an effective intervention target for the treatment of NDs needs to be further verified through a large number of experiments; (2) The precise mechanisms of specific gut microbes and their metabolites in NDs remain uncharacterized; (3) Molecular pathways by which CHM interventions affect the gut microbiota are undefined; (4) CHM also has problems such as complex components, difficulty in large-scale purification of single active components, and low bioavailability. Using molecular imprinting technology as adsorbents not only ensures that precious CHM products are not wasted, but also realizes the quality detection of precious CHM products, which is of great significance for the quality control and safe drug use of precious CHM products. Moreover, a novel Dual Graph Attention Network designed to predict CHM drug-drug interactions by extracting key structural features of active molecules within the herbal ingredients; (5) Current evidence is predominantly derived from preclinical animal studies; while promising, the therapeutic efficacy of CHM targeting the gut-brain axis remains to be further validated through large-scale, rigorously conducted clinical trials in human subjects. (6) Current research mainly focuses on empirical or self-designed CHM prescriptions, with insufficient randomized controlled trials validating classical prescriptions. Therefore, future studies of CHM in the treatment of NDs through regulating the gut microbiota can be carried out by comprehensively using genomics, proteomics, transcriptomics, metabolomics, and microbiomics. For example, a multi-omics approach, including transcriptomics, gut microbiota analysis, and metabolomics, was employed to elucidate the protective effect of resveratrol hydroxypropyl-β-cyclodextrin inclusion complex against PD by regulating the microbiota-gut-brain axis (6) Current research mainly focuses on empirical or self-designed CHM prescriptions, with insufficient randomized controlled trials validating classical prescriptions. Therefore, future studies of CHM in the treatment of NDs through regulating the gut microbiota can be carried out by comprehensively using genomics, proteomics, transcriptomics, metabolomics, and microbiomics. For example, a multi-omics approach, including transcriptomics, gut microbiota analysis, and metabolomics, was employed to elucidate the protective effect of resveratrol hydroxypropyl-β-cyclodextrin inclusion complex against PD by regulating the microbiota-gut-brain axis [124]. Integrating multi-omics (proteomic and metabolomic) revealed that danggui-shaoyao-san decoction improved brain glucose uptake and mitigated mitochondrial dysfunction and oxidative stress by regulating the GSB3β/PGC1α pathway in AD [125]. Chen *et al*. [126] reported that integrating multi-omics contributed to clarifying the role of the microbiota-gut-brain axis in ND. Solving the above problems will help clarify the biological mechanisms by which CHM prevents and improves NDs, and provide new perspectives for clinical treatment and drug development.” to “(6) Current research mainly focuses on empirical or self-designed CHM prescriptions, with insufficient randomized controlled trials validating classical prescriptions. Therefore, future studies of CHM in the treatment of NDs through regulating the gut microbiota can be carried out by comprehensively using genomics, proteomics, transcriptomics, metabolomics, and microbiomics. Solving the above problems will help clarify the biological mechanisms by which CHM prevents and improves NDs, and provide new perspectives for clinical treatment and drug development.

In summary, targeting the gut microbiota by CHM represents a promising novel strategy for the prevention and treatment of NDs. CHM could be used as a monotherapy or combined with other therapeutics to achieve disease intervention in NDs. Combining the promising potential of CHM with their molecular mechanisms and techniques could remarkably aid in the development of new treatments for NDs.

## Supplemental Materials

Supplementary data for this paper are available on-line only at http://jmb.or.kr.



## Figures and Tables

**Table 1:** Dysbiosis of gut microbiota in neurodegenerative diseases from 2021 to 2025.

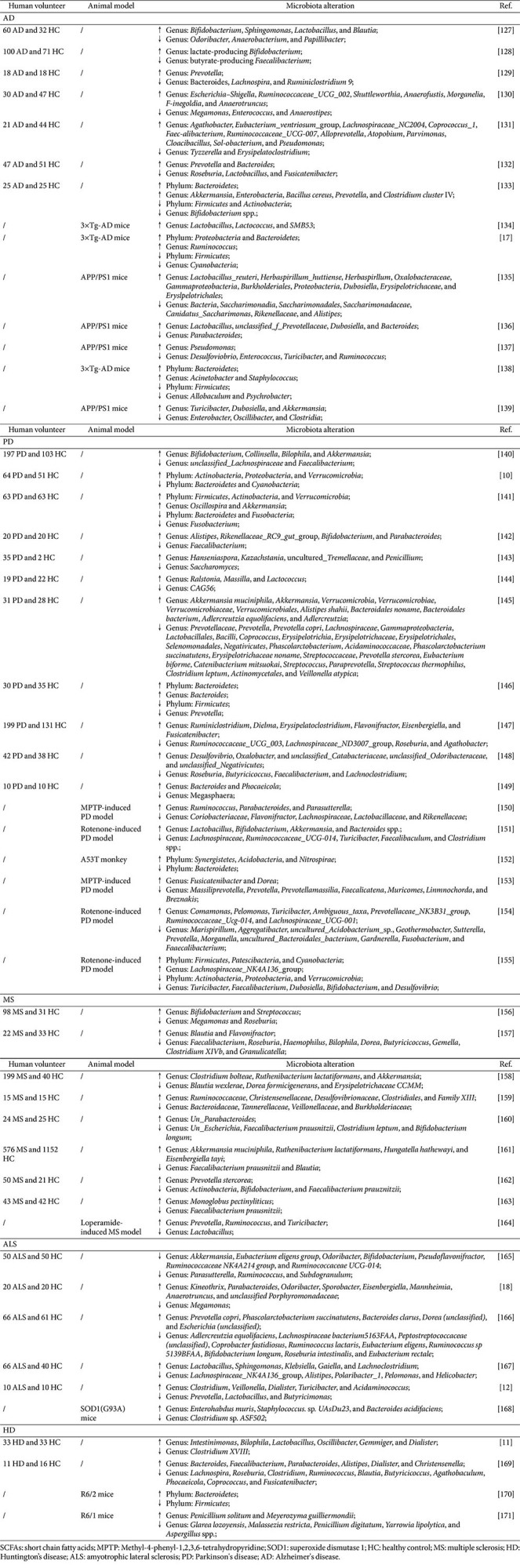

**Table 2:** Effects of Chinese herbal medicine treatments on gut microbiota in animal models of neurodegenerative diseases from 2021-2025.

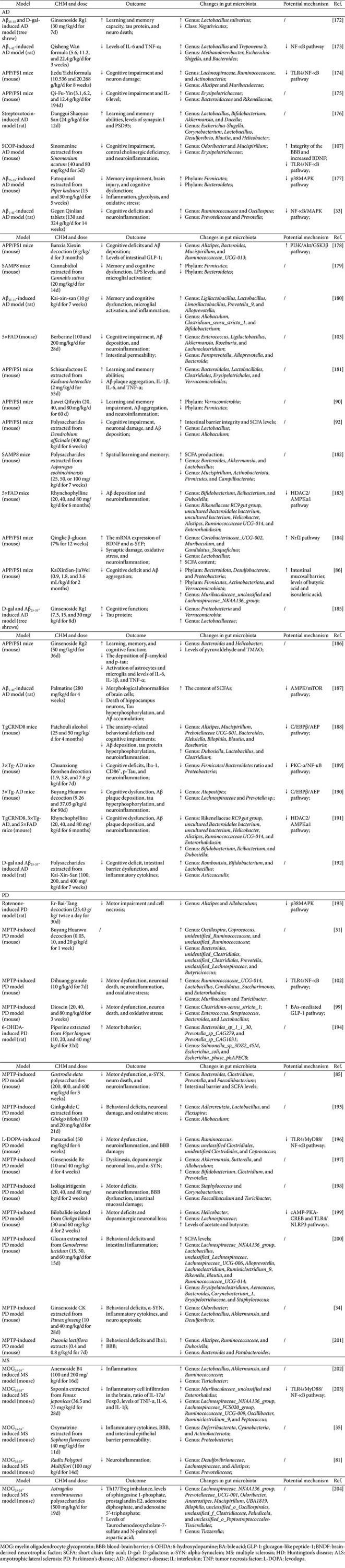

**Table 3:** Clinical trials of traditional Chinese medicine in neurodegenerative diseases