# Supplementary material for: Chinese Herbal Medicine Targets Gut Microbiota to Combat Neurodegenerative Diseases: Potential Mechanisms and Clinical Implications
Source: J Microbiol Biotechnol. 2026 Feb 25;36:e2510008. doi: 10.4014/jmb.2510.10008 (PMC12975504; doi:10.4014/jmb.2510.10008)
Supplement: Supplementary file 1 [file jmb-36-e2510008-supple.pdf]

J. Microbiol. Biotechnol. 2026. 36: e2510008
https://doi.org/10.4014/jmb.2510.10008

Supplementary Table S1. Clinical trials of traditional Chinese medicine in neurodegenerative diseases

Category	Year	Sponsor	Clinical trial ID
AD			
Wujia Yizhi granules	2024	Sichuan Jishengtang Pharmaceutical Co., Ltd., China	NCT06534723
Smart soup<br>(Consisting of *Acori Tatarinowii* Rhizoma, *Wolfiporia cocos* (Schw.) Wolf (Poria)., and *Radix Polygalae*)	2022	Peking Union Medical College Hospital, China	NCT05538507
Yangxue Qingnao pills<br>(Consisting of *Angelicae sinensis* Radix, *Chuanxiong* Rhizoma, *Paeoniae Radix* Alba, *Rehmannia glutinosa*, *Uncaria macrophylla* Wall, *Caulis spatholobi*, *Spica Prunellae*, *Catsia tora* Linn, *Mater Margarita*, *Corydalis ambigua*, and *Asarum sieboldii*)	2021	Dongzhimen Hospital, China	NCT04780399
VGH-AD1<br>(Consisting of Yi-Gan San, Huan-Shao Dan, *Radix et Rhizoma salviae Miltiorrhizae*, *Rhizoma gastrodiae*, *Ramulus Uncariae cum Uneis*, and *Radix Morindae officinalis*)	2020	Taipei Veterans General Hospital, Taiwan	NCT04249869
Flos gossypii flavonoids	2020	Capital Medical University, China	NCT05269173
Huannao Yicong formula<br>(Consisting of *Polygonum multiflorum*, *Panax ginseng*, *Acorus tatarinowi*, *Coptis chinensis*, and *Ligusticum chuanxiong*)	2017	Xiyuan Hospital, China Academy of Traditional Chinese Medicine, China	ChiCTR-IOR-17011746
Yishen Huazhuo decoctions<br>(Consisting of *Epimedium brevicornum* Maxim., *Fructus Ligustri Lucidi*, *Radix Polygoni Multiflori*, *Cistanche sinensis* Beck, *Ligusticum wallichi* Franchat, and *Acorus gramineus*)	2012	Tianjin university of traditional Chinese medicine, China	ChiCTR-TRC-12002846
Di-Tan decoctions	2012	Hong Kong Baptist University,	ChiCTR-TRC-12004548

(Consisting of *Arisaema Cum* Bile, *Pinelliae Rhizoma*, *Aurantii Immaturus* Fructus, *Poria*, *Citri Reticulatae Pericarpium*, *Acori Tatarinowii* Rhizoma, *Ginseng Radix*, *Taeniam Caulis*, *Glycyrrhizae Radix*, *Zingiberis Recens* Rhizoma, and dextin)		China	
PD			
Tianqi Pingchan granules (Consisting of *Astragalus mongholicus* Bunge, *Rehmannia glutinosa* (Gaertn.), DC, *Paeonia lactiflora* Pall, *Angelica sinensis* (Oliv.) Diels, Uncaria rhynchophylla (Miq.) Miq, *Gastrodia elata* Blume, and *Bombyx batryticatus*)	2020	Xinhua Hospital, Shanghai Jiao Tong University School of Medicine, China	NCT04173832
SQJZ herbal mixtures (Consisting of *Rehmannia glutinosa*, *Cornus officinalis*, *Ophiopogon japonicus*, *Poria cocos*, *Trichosanthes kirilowii*, *Cuscuta chinensis*, *Semen ziziphin spinosae*, *Schisandra chinensis*, and *Aurantii fructus immaturus*)	2015	Dongzhimen Hospital, China	NCT02616120
Lingzhi (*Ganoderma lucidum*)	2005	Xuanwu Hospital, China	NCT00224263
Wuling capsules (Consisting of *Xylaria nigripes* (Kl.) Sacc.)	2021	Xinhua Hospital, Affiliated to Shanghai JiaoTong University, China	ChiCTR2100046195
Zishen pingchan granules (Consisting of *Radix Rehmanniae* preparata, *Lycium barbarum*, *Herba Taxilli*, *Rhizoma gastrodiae*, *Stiff silkorm*, *Curcuma phaeocaulis*, *Radix Paeoniae* Alba, *Rhizoma arisaematis*, *Opistophthalmus ecristatus*, and *Scolopendra subspinipes* mutilans)	2018	The Affiliated Brain Hospital of Nanjing Medical University (Nanjing Brain Hospital), China	ChiCTR1800019942
Pingchan granules (Consisting of *Lycium barbarum* l., *Taxillus chinensis* (DC.) Danser, *Gastrodia elata* Blume, *Paeonia lactiflora* Pall., *Arisaema erubescens* (Wall.) Schott, *Curcuma phaeocaulis* Valeton, *Bombyx mori* Linnaeus, *Buthus martensii* Karsch, and *Scolopendra subspinipes* mutilans l. Koch)	2017	Longhua Hospital Affiliated to Shanghai University of Traditional Chinese Medicine, China	ChiCTR-INR-17011949
Congrong Shujing granules (Consisting of *Cistanche deserticola* Y.C. Ma, *Polygonatum kingianum* Collett & Hemsl., *Salvia miltiorrhiza* Bunge, *Paeonia lactiflora* Pall., and *Paeonia suffruticosa*	2016	Fujian University of Traditional Chinese Medicine, China	ChiCTR-IOR-16008394

Andrews)<br>Xifeng Dingchan pills<br>(Consisting of *Reynoutria multiflora* (Thunb.) Moldenke, *Carapax Testudinis*, *Gastrodia elata* Blume, *Bombyx mori* L., *Acorus gramineus* Aiton, *Conioselinum anthriscoides*, and *Paeonia lactiflora* Pall.)	2012	The First Affiliated Hospital of Henan University of Traditional Chinese Medicine, China	ChiCTR-TRC-12002150
ALS			
Traditional Chinese medicine<br>(including Chinese herbal medicine, acupuncture, moxibustion, massage, taiji, and qigong)	2021	Dongzhimen Hospital, China	NCT04885374

Note: The description clarifies: (1) Decoctions (soups): Aqueous extracts of raw herbal materials, prepared by boiling and simmering; (2) Granules: Concentrated and lyophilized powder of herbal decoctions, for convenient reconstitution; (3) Mixtures: Homogenized liquid preparations of multiple herbal extracts; (4) Formulas: Combinations of two or more herbal materials formulated based on TCM syndrome differentiation principles.
